# Numbers, systems, people: how interactions influence integration. Insights from case studies of HIV and reproductive health services delivery in Kenya

**DOI:** 10.1093/heapol/czx097

**Published:** 2017-11-24

**Authors:** Susannah H Mayhew, Sedona Sweeney, Charlotte E Warren, Martine Collumbien, Charity Ndwiga, Richard Mutemwa, Irina Lut, Integra Initiative, Manuela Colombini, Anna Vassall

**Affiliations:** 1 Department of Global Health and Development, Faculty of Public Health and Policy, London School of Hygiene and Tropical Medicine, London, UK; 2 Population Council, Washington, DC, USA; 3 Department of Social and Environmental Health Research, Faculty of Public Health and Policy, London School of Hygiene and Tropical Medicine, London, UK; 4 Population Council, Nairobi, Kenya; 5 Centre for Infectious Disease Control – Zambia (CIDRZ), Zambia; 6 Family Planning Association, UK; 7 Full list of Integra Initiative team members is provided in the Acknowledgements

**Keywords:** Integration, health systems, evaluation, reproductive health, HIV

## Abstract

Drawing on rich data from the Integra evaluation of integrated HIV and reproductive-health services, we explored the interaction of systems hardware and software factors to explain why some facilities were able to implement and sustain integrated service delivery while others were not. This article draws on detailed mixed-methods data for four case-study facilities offering reproductive-health and HIV services between 2009 and 2013 in Kenya: (i) time-series client flow, tracking service uptake for 8841 clients; (ii) structured questionnaires with 24 providers; (iii) in-depth interviews with 17 providers; (iv) workload and facility data using a periodic activity review and cost-instruments; and (v) contextual data on external activities related to integration in study sites. Overall, our findings suggested that although structural factors like stock-outs, distribution of staffing and workload, rotation of staff can affect how integrated care is provided, all these factors can be influenced by staff themselves: both frontline and management. Facilities where staff displayed agency of decision making, worked as a team to share workload and had management that supported this, showed better integration delivery and staff were able to overcome some structural deficiencies to enable integrated care. Poor-performing facilities had good structural integration, but staff were unable to utilize this because they were poorly organized, unsupported or teams were dysfunctional. Conscientious objection and moralistic attitudes were also barriers.

Integra has demonstrated that structural integration is not sufficient for integrated service delivery. Rather, our case studies show that in some cases excellent leadership and peer-teamwork enabled facilities to perform well despite resource shortages. The ability to provide support for staff to work flexibly to deliver integrated services and build resilient health systems to meet changing needs is particularly relevant as health systems face challenges of changing burdens of disease, climate change, epidemic outbreaks and more.

Key Messages

- Most evaluations of service and health systems integration focus on the structural dimension: physical infrastructure and resources; trained staff; service statistics.
- Integra demonstrates that structural integration (of infrastructure, supplies, trained staff) does not necessarily lead to integrated service delivery.
- Structural factors can be influenced, and overridden, by frontline and management staff who hinder or achieve functional integration. Key facilitators are the existence of agency among frontline staff, flexible, team-approaches to load-sharing and supportive management.
- The ability to provide an integrated service to meet changing needs is particularly relevant as health systems face changing and unpredictable burdens of disease as a result of climate-change and double/triple-burdens of non-communicable, infectious and chronic diseases.

## Introduction

Debate on the advantages and disadvantages of integrated health care versus vertical programming has persisted since Alma Ata (WHO 1978). In the field of sexual and reproductive health integration has been increasingly promoted in low-income settings dominated by ‘vertical’ health programmes as a means to bring together related services to improve their efficiency and efficacy (UNFPA 2004; WHO and UNFPA 2006; WHO/UNFPA 2017; and see Warren *et al.* (2017) in this Edition for a historical review). In high-HIV prevalence settings in sub-Saharan Africa, concern grew in the 2000s to improve access to HIV testing and treatment services through mainstream, as well as HIV-specialist, health facilities. Additionally, integration of HIV with other health services was seen as an important mechanism to strengthen health systems (Coovadia and Bland 2008) and improve efficiency and holistic care (meeting individual needs), as well as increasing uptake of services and patient outcomes (Church and Mayhew 2009; Sibide and Buse 2009; Kennedy *et al.* 2010).

Nevertheless, the evidence for the improvements hypothesized from integrating HIV and other SRH services has been patchy (Dudley and Garner 2011; Lindegren *et al.* 2012; Wilcher *et al.* 2013) and a growing body of literature suggests challenges in implementing integration have impeded its delivery and subsequent improvements in health outcomes. Research highlights, e.g. deficiencies in the capacity and willingness of providers to deliver a broader package of care particularly where working conditions are poor (Mutemwa *et al.* 2013; Uebel *et al.* 2013), reluctance of providers to move beyond routine practices or take on new roles (Shelton 2001; Reeves *et al.* 2010; Smit *et al.* 2012). Health systems barriers including infrastructure, equipment, data management, managerial and human resource factors (Church and Mayhew 2009; Uebel *et al.* 2013; Topp *et al.* 2013; Wilcher *et al.* 2013), are also recorded.

In lower- and middle-income contexts integration is usually understood as the amalgamation of previously separate components of care, or the addition of a new intervention into an existing service (e.g. adding HIV testing to FP services) (Criel *et al.* 1997; Ekman *et al.* 2008; Dudley and Garner 2011). There is no standard definition of ‘integration’, however, and even HIV-SRH integration is variously defined in different studies (Fleischman *et al.* 2002; Maharaj and Cleland 2005; Mutemwa *et al.* 2013; Mayhew *et al.* 2016). Nevertheless, it is widely recognized in the literature that conjoining two previously separate services involves consideration of infrastructure, staff training, management and supervision structures and logistics and supplies. As such, integration of services is seen as a complex public health intervention. A growing body of health systems scholarship has identified two elements that are distinguishable that affect interventions: the available systems ‘hardware’ (equipment, infrastructure, trained staff) and systems ‘software’ (the values, attitudes and practices of the staff responsible for delivering and managing integrated services) within which there is growing attention to the notions of ‘trust’ and ‘power’ (Gilson *et al.* 2005, 2011; Gilson 2006; Erasmus and Gilson 2008; Sheikh *et al.* 2011). Yet, evaluations have tended to focus more on the hardware than software components and few analyses understand how hardware and software components interplay.

The Integra Initiative (Integra) (described below) is the largest evaluation trial for integrated HIV and RH services globally. It sought to evaluate the impacts of different models of integrating HIV testing and treatment services with mainstream family planning (FP) and post-natal services in Kenya and Swaziland (Warren *et al.* 2012). Like most trials it reports on impact, but with an important difference. Integra understands and measures integration as a continuum from separately managed and delivered programmes (e.g. HIV programmes and FP programmes) to full integration of infrastructure (multi-use rooms, multi-trained staff, joint procurement and supply chains, integrated management etc.) and care, recognizing that the degree of integration implemented is not the same in any two health facilities (Mayhew *et al.* 2016). This gives a more nuanced and robust understanding of the impact of integrated services and health facilities over time since it directly addresses the issue of confounding caused by the reality that the implementation of ‘integrated services’ is not homogenous but varies widely between facilities. The Integra Index was developed using facility-specific data to calculate a facility-specific integration measure (Mayhew *et al.* 2016). The Index was then used to analyse impact; Integra found, at an individual level, that exposure to integrated facilities had a positive effect on HIV testing among clients (Church *et al.* 2017). At a process level integrating HIV testing and care services also seems to have a positive effect on technical quality of care for the host service (FP) (Mutemwa *et al.* 2017 reported in this supplement). Integra cost findings showed that integration has the potential for workload and cost-efficiencies, but these are often not realized (Sweeney *et al*. 2014; Obure *et al*. 2015).

The remaining piece of the integration jigsaw was to understand what factors influence relative success in delivering integrated services. This paper draws on Integra’s rich mixed methods data to unpick the ‘how’ of integration and explore the interaction of systems hardware, software and contextual factors to explain why some facilities appeared to be able to implement and sustain integrated service delivery while other similar facilities did not.

## Methods

### The Integra Initiative

Integra’s goal was to strengthen the evidence of the benefits and costs of a range of models for delivering HIV services integrated with FP and postnatal care (PNC) services in high-prevalence (Swaziland) and medium-prevalence (Kenya) HIV settings. The study originally sought a controlled, non-randomized intervention design to measure the effect of integrated health care (Warren *et al.* 2012). Facilities were assigned, in consultation with the Ministry of Health, to intervention or comparison arms of the study. Intervention facilities received equipment, training on a service-delivery algorithm and a mentorship programme.

The study intervention is described in detail elsewhere (Warren *et al.* 2012), but in short it was implemented between 2009 and 2011 and was designed, in Kenya, to add the following services into standard FP service delivery: discussion of fertility desires, condom promotion/provision, STI/HIV risk assessment, HIV status check, HTC provision, cervical cancer screening, pre-HIV treatment services and/or referral to HIV treatment unit for HIV+ clients. The provision of these services was supported by training on and the provision of an integrated client counselling toolkit, the ‘Balanced Counseling Strategy Plus’ (Population Council 2016). In addition, intervention facilities were supported by nurse/midwife ‘mentors’ who were trained as mentors and provided training on SRH/HIV technical skills and supportive supervision on integrated care (see Ndwiga *et al.* 2014 for details). The layout of some Facilities was also reorganized to support integrated care provision, and essential equipment and supplies were provided to deliver integrated services. By agreement with the Government of Kenya, initial clinical supplies and equipment (including autoclaves) were provided to study facilities to ensure some degree of equity between them at the start of the study. After this, routine government medical supply systems took over (by early 2010). Throughout the study there was regular contact with the MoH.

**Figure 1:**
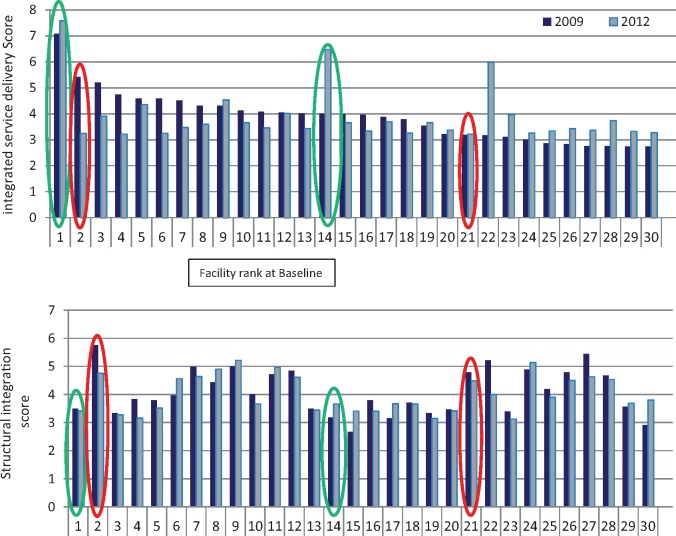
Integra index rankings for Kenya

In Kenya, however, during the trial the government formally adopted and accelerated implementation of integrated HIV and SRH services in all public health facilities (by early 2011). This, together with actions by individual Facility managers, NGOs and external donors, removed operational distinction in service provision between facilities in intervention and comparison arms. Consequently, assessment of the primary outcome was shifted from comparison of study arms to comparison of individual facilities depending on the level of integration each achieved through the study. A facility’s ‘level’ of integration was measured by an ‘integration index’ which gives a relative ranking of Facilities at four timepoints through the study based on aggregate, model-weighted data from a range of indicators (see Mayhew *et al.* 2016 for details). The results for Kenya (shown in Figure 1) were used to identify four case-study facilities for analysis.

### Definition of integration

Integra explicitly defined service integration as the provision during one visit of any reproductive health service [defined as FP, antenatal care (ANC), PNC] AND any HIV/sexual health-related service (defined as HIV counselling and testing, HIV anti-retroviral therapy (ART) treatment, CD4 count services, STI treatment, cervical cancer screening).

Two dimensions were investigated: ‘structural’ and ‘functional’ integration (described in Mayhew *et al.* 2016). These and the five sets of data used are summarized in the Table 1 and are described in detail in the text that follows.

**Table 1:** Summary of methods and contribution of each to systems analysis

Data Source	Description	Indicators	Contribution to systems analysis
Staffing and facility data Collected in all facilities 2009–11	Periodic Activity Review collected data on staff time available and how much time was spent on each of the services of interest^a^ A service was assessed as ‘available’ if more than 10 visits were recorded in a year.	Service availability at MCH/FP^b^ Unit: % of HIV-related services [1–5 below^a^] available in the MCH/FP unit at each facility. Service availability at facility: % of RH [6–8 below^a^] and HIV-related services available anywhere in the facility	Structural hardware data on staffing time available for each of the services being integrated.
	Data from facility registers in all facility 2009–11	Range services per room: % HIV-related services that are provided in each MCH/FP consultation room. Range of services per provider: % HIV-related services that are provided per MCH/FP clinical staff member in a day	Structural hardware data on room use and provider care, measuring how integrated they are.
Time-series client flow data 2010–12: 8841 clients tracked across six timepoints in the four case-study facilities.	Captures service utilization patterns, and receipt of integrated services, among clients seeking MCH-FP services. Receipt of integrated services defined as: (1) a client received any HIV or STI service, specifically: HIV testing, counselling or treatment; PMTCT; STI counselling or testing; cervical cancer screening AND (2) any of the following MCH services: FP counselling or provision; PNC for mother or baby; ANC.	Range of services accessed daily: % days in the week on which any RH services [6–8 below] AND any HIV-related services [1–5 below] are accessed Range of services provided in one consultation: % clients who receive any RH services AND any HIV-related services in one of their provider contacts Range of services provided in one visit to facility: % who receive any RH services AND any HIV-related services during their visit to the facility (1 day) HIV treatment location and referral^c^: location of ART and functionality of referral system to ART for SRH clients	Outcome data used to verify whether integrated services are received and patterns of receipt over time. The Integra Index defines this as ‘functional’ integration. The achievement (or not) of functional integration (i.e. clients actually receiving integrated care) is then interpreted in the light of analysis of the interplay between hardware and software factors.
Structured provider interviews 24 providers in late 2011/early 2012	Views and experiences (including Likert-scale questions) of providers with integrated service delivery, perceived benefits and challenges and information on supervision and facility management.	N/A	Both structural hardware data on training and capacity of staff and software data on provider perceptions of what factors (e.g. infrastructure, support) are important in helping them deliver ‘functional’ integrated care.
Qualitative in-depth provider interviews 17 providers between 2010 and 2013	Experiences of frontline health care providers of implementing integration and exploration of facilitators and challenges.	N/A	Provides insights into what ‘soft’ or less quantifiable factors (like team support) front-line providers feel are important for enabling integrated delivery of care (i.e. achievement of functional integration).
*Context data*	Standard tool recording: donors, NGOs and other players active at the facility in HIV and RH; details of the activities being carried out and how; staffing levels, redeployment or new staff, new infrastructure and commodities status over time.	N/A	Influence of external factors on structures, infrastructure, supplies etc. on ability to deliver integrated services.

a Eight services were assessed: HIV-related services are (1) ART; (2) cervical cancer screening; (3) CD4 count services; (4) HIV/AIDS testing services; (5) STI treatment. RH services are (6) FP; (7) PNC; (8) ANC.

b Maternal and child health/FP unit.

c We recognized that the appropriateness of including this indicator is dependent on the need for ART in the catchment population; we took into account the fact that smaller clinics do not provide ART on site, by recognising referrals.

The dimensions of ‘structural’ and ‘functional’ used here were defined during the Integra Index analysis (Mayhew *et al.* 2016) in which the nature and degree of service-integration (as defined earlier) at health facility level was investigated through modelling of facility level data across the 30 study sites in Kenya: 24 public health facilities and 6 NGO facilities selected in Central and Eastern Provinces^1^ as well as 12 further sites in Swaziland. This analysis showed that ‘structural’ and ‘functional’ integration are two distinct and uncorrelated dimensions of integration operating at facility level. The analysis defined structural integration as measurable elements of infrastructure, multi-trained staff etc. at each facility (not higher level Ministry programmes); functional integration was defined as integrated receipt of care by a client at that facility. The analysis suggested that the existence of structural integration at a facility was not sufficient in and of itself to achieve functional integration. The purpose of the present paper was to investigate this further through a better understanding of the structural and functional dimensions and why one might not be sufficient to lead to the other, despite common assumptions that once structural components are in place to support integration, integrated service delivery will follow. The factors associated with structural integration are closely akin to those commonly described in the health systems literature as ‘systems hardware’. The notion of functional integration has no direct correlate being in a sense an outcome measure (integrated care actually delivered). Nevertheless, a hypothesis which we pursue is that the achievement of functional integration (which in the Integra Index is not correlated with the existence of structural integration) is heavily dependent on systems ‘software’ factors including provider motivation and morale.

### Data sources for this article

This article reports in detail on four case-study facilities drawn from the total sample of 30 facilities in Kenya.

#### Staffing and facility data

A detailed description of the data collection and analysis of workload data is provided elsewhere (Sweeney *et al.* 2014) but in brief this component involved collecting data on facility organization, staff time and workload using two instruments: a semi-structured interview and records review tool (constituting a Periodic Activity Review) and a costing instrument. Both instruments were pretested in field sites and revised before implementation. Data were collected at baseline (2008–09) and endline (2010–11) in all 30 study Facilities.

A mixed methods approach assessed available staff time: key informant interviews with staff, followed by 1 week of direct observation by researchers, time consultations and concurrent time sheets completed by facility staff members. Finally, a confirmatory interview with the staff member at the end of the observation period discussed any discrepancies between data sources. Data were entered into standardized Excel worksheets, and analysed using Excel (Microsoft Corp., Redmond, WA, USA) and Stata 13.

Service availability at each facility was assessed through interviews with service providers, and confirmed using service statistics. Services were regarded as ‘available’ within a facility if more than ten visits were recorded in a year—this was intended to assess whether a facility had the capacity to deliver services; ten was selected to exclude facilities that simply mis-coded a few visits.

#### Time-series client flow data

Client flow assessments (CFAs) were designed to capture service utilization patterns, and receipt of integrated services, among clients seeking MCH-FP services and are discussed in detail elsewhere (Birdthistle 2014).

CFAs were implemented as time-series (six times from June 2009 to February 2012). Over a period of 5 days, Monday–Friday, all clients entering the facility for MCH-FP services were given a client flow form by trained local researchers or service providers. Clients carried the form throughout their visit, and each service provider they saw completed the form in their consultation room, indicating session start/end times, service(s) received by the client and any referrals to other providers.

A total of 8841 visits were tracked across the four case-study facilities in Kenya. A ‘visit’ (the unit of analysis) comprised all providers seen and services received in the same day for each client, as captured on the client assessment form. Clients were either a single adult (male or female) or an adult plus a child. An ‘integrated service’ was deemed to have been received where (1) a client received any HIV or STI service, specifically: HIV testing, counselling or treatment; PMTCT; STI counselling or testing; cervical cancer screening AND (2) any of the following MCH services: FP counselling or provision; PNC for mother or baby; ANC.

#### Structured provider interviews

Our article utilizes results of structured survey interviews conducted in each of the study facilities in late 2011/early 2012. Provider interviews were conducted using consecutive sampling, with the next available health worker in the study facility: a total of 24 interviews were conducted at the four case-study facilities analysed in this article. Written informed consent was obtained from each respondent. Interviews covered views and experiences of providers with integrated service delivery, perceived benefits and challenges and information on supervision and facility management. They asked specific questions on whether team-work and communications had improved. They also included Lickert-scale statements developed after initial analysis of the first round of qualitative provider interviews (e.g. asking staff if they strongly/agreed or strongly/disagreed with the statement ‘Staff work together much more now than previously’). These statements further explored software issues of whether staff felt supported in their jobs and what challenges, as well as enablers, staff experienced. All interviews were conducted in English and analysed using Stata 11.2.

#### Qualitative provider interviews

Interviews covered experiences of frontline health care providers in the selected hospitals, sub-district hospitals and health centres in each study district. A total of 17 in-depth semi-structured interviews were conducted with health care providers: 10 in June/July 2010 and 7 (mainly managers) in May 2013. In each facility, a senior manager was identified (in some there was only one) and front-line providers on duty during the day of the field visit were approached. One facility (1) was included for in-depth interviews only in 2013, having been identified later as a facility of interest; the visit prioritized the manager interview as front-line staff had already been surveyed. Although in other facilities front-line staff were additionally interviewed in depth none of them were available from this facility at the time of the field visit as they were too busy, which may be a reflection of the fact that they prioritized their clients rather than researchers. Time and resource constraints did not allow repeat visits for this component. The purpose of the interviews was for providers to reflect on the previous years of the study, including how integration is managed in their facilities; their experiences of how they were able (or not) to work together to deliver integrated care and an exploration of facilitators and challenges. Each respondent provided written informed consent to participate in the study and be interviewed. Interviews were conducted in English or Kiswahili (at the choice of the interviewee) by two trained facilitators. Transcripts were double coded by RM and MC and managed using Nvivo 8; thematic analysis was used. More detail on these methods and the results from the qualitative provider interviews across the Integra sites in Kenya is provided in Mutemwa *et al.* (2013).

#### Context data

Throughout the study period a record was kept by our partners on the ground of the external influences in the study facilities and in the catchment area of each facility (e.g. donor activities or government campaigns in the facility/facility catchment areas).

A standard tool was developed to record: donors, NGOs and other players active at the facility in HIV and RH; details of the activities being carried out and how; staffing levels, redeployment or new staff, new infrastructure and commodities status over time. To document this, project staff visited each site at least twice during the project period. Data were obtained from observations and talking to the facility managers.

### Study limitations

The biggest limitation to this study is the variation in qualitative data between the case study sites. In particular Facility 1 only has one in-depth staff interview (as well as seven structured interviews with providers), compared with between four and seven in the other sites. Also, client data do not form part of this paper, though findings on client perspectives of integrated service delivery have been published elsewhere (Colombini *et al.* 2016).

## Selection of facility case studies

A key finding of the Integra Index analysis (Mayhew *et al.* 2016) was that ‘structural integration’ (i.e. resources and staff—akin to systems hardware) is not correlated with ‘functional integration’ (i.e. actual delivery of integrated care) (Table 1). A further finding was that there is a diversity of patterns across facilities over time. We therefore looked at changes in the baseline-endline ranking of the 30 Kenyan facilities generated by the Integra Integration Index to identify high and low performing integrated facilities (Figure 1). We selected a range of facilities for detailed case-study analysis that showed contrasting trends in order to explore why some facilities are able to sustain integrated performance over time, even with poorer structural scores, and better understand how systems hardware and software factors interact. Figure 1 shows the ranking of facilities according to their functional integration scores (top panel) and structural integration scores (lower panel).

Facility 1 is clearly an outlier in terms of consistent high-ranking functional integration performance (high-high performance). Facility 14 was selected for its very significant functional integration improvement (low-high performance). Both facilities show considerable contrast with their relatively lower structural integration ranks (lower panel). Facility 2 shows the biggest decline in functional integration over time despite very good structural integration (high-low performance). Facility 21 was selected as an example of a consistently poor performer functionally, despite quite good structural integration (low–low performance).

Once the facilities were selected we looked at the disaggregated indicators of ‘functional integration’ and quantitative facility data to compare facilities. We then analysed detailed contextual and qualitative interview data by facility to examine what appeared to explain the sustained or improving functional-integration ranking of some facilities over time compared with others.

## Results and discussion

### Case study facilities

The characteristics of the four case facilities are summarized in Table 2 and discussed in detail in the ‘realities’ section below. Before this, we describe macro-level differences in the performance of each facility which the case studies help to interpret.

**Table 2:** Overview of case study facilities: services, client visits, staff and organization of integration

Facility ID	Type and location	Services offered^a^	Mean Annual Visits^a^		Mean staff available (FTE)^a^		HIV testing and treatment integration^b^	Data available
			2009	2011	2009	2011		
**Kenya**								
Facility 1: High to High Eastern Province	Sub-District Hospital (since 2010) Rural 14 Bed	FP	1028	2015	0.68	0.98	2009: PITC in FP and ANC (PMTCT) 2010: PITC in all Departments (no stand-alone VCT) Treatment in CCC); for HIV+ pregnant women they received full HIV care and maternal/6 m post-partum care in MCH Unit.	Staffing and facility data 2009, 2011 Client Flow data (Jun/Jul 2009; Jan 2010; June 2010; Jan/Feb 2011; Aug 2011; Jan 2012) 7 Structured provider interviews, 2011/12 1 In-depth provider interview 2013 Context data
		Ca Cervix screening		24		0.01		
		STI						
		HIV care and Tx	154	3092	0.07	6.27		
		PITC	102	685	0.07	0.35		
		VCT	109		1.02			
		Other MCH/ANC	4426	4479	8.07	8.40		
		TOTALS	5819	10 295	9.91	16.01		
		Catchment population	N/A	12 800				
Facility 2: High to low Central Province	Health Centre Peri-urban	FP	2276	1644	1.42	0.66	PITC in FP (initially each visit; now ‘when necessary’). Also group counselling and testing with individaul results-discussion. Treatment in separate CCC—no FP provided; pregnant WLHIV (7 at endline) are referred to MCH/FP but don’t necessarily receive full care there	Staffing and facility data 2009, 2011 Client Flow data (Jun/Jul 2009; Jan 2010; June 2010; Jan/Feb 2011; Aug 2011; Jan 2012) 6 Structured provider interviews, 2011/12 5 In-depth provider interviews (3 in 2010; 2 in 2013) Context data
		Ca Cervix screening		342		0.30		
		STI	128		0.01			
		HIV care and Tx						
		PITC	241	518	0.15	0.65		
		VCT	483		1.00			
		Other MCH/ANC	868	2379	12.47	11.15		
		TOTALS	3996	4783	15.05	12.76		
		Catchment population	23 000	93 000				
Facility 14: Low to High Central Province	Health Centre Peri-urban 4 bed	FP	2472	1572	0.98	4.47	No stand alone VCT; PITC in MCH (PMTCT), FP and OPD. FP repeat clients test every 3 m HIV+ results mean clients are linked to STI, ART and support services. Treatment in CCC provided 1 day/week in OPD (fully integrated incl FP provision)	Staffing and facility data 2009, 2011 Client Flow data (Jun/Jul 2009; Jan 2010; June 2010; Jan/Feb 2011; Aug 2011; Jan 2012) 5 Structured provider interviews, 2011/12 4 In-depth provider interviews (2 in 2010; 2 in 2013) Context data
		Ca Cervix screening		68		0.33		
		STI						
		HIV care and Tx						
		PITC	1385	876	0.55	0.33		
		VCT						
		Other MCH/ANC	6450	2388	2.57	3.52		
		TOTALS	10 307	4904	4.10	8.65		
		Catchment population	40 000	7684				
Facility 21: Low to Low Central Province	Sub-District Hospital (since 2008) 24 Bed Peri-urban	FP	2447	3348	0.90	0.87	Group counselling and testing for FP/MCH clients (self-reading of results) CCC is considered to lack privacy and is not well used.	Staffing and facility data 2009, 2011 Client Flow data (Jun/Jul 2009; Jan 2010; June 2010; Jan/Feb 2011; Aug 2011; Jan 2012) 6 Structured provider interviews, 2011/12 7 In-depth provider interviews (5 in 2010; 2 in 2013) Context data
		Ca Cervix screening	26	30	0.01	0.05		
				
		STI						
		HIV care and Tx						
		PITC	250	230	0.09	0.06		
		VCT	275		0.33			
		Other MCH/ANC	3900	2167	3.88	20.44		
		TOTALS	22 198	5745	5.21	21.42		
		Catchment population	N/A	120 000				

a Data source, Periodic Activity Reviews 2009–10 and 2010–11.

b Interviews and Observations undertaken for the staffing and facility assessment.

### Facility comparisons: availability and performance of delivering integrated care

**Figure 2:**
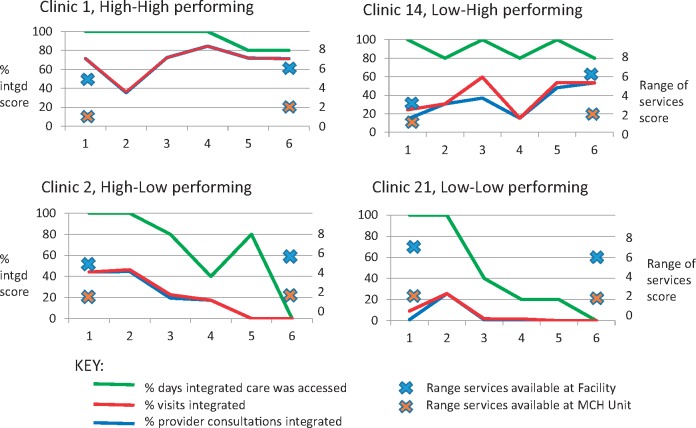
Capacity-delivery gap in case study facilities

Figure 2 shows three core indicators of ‘functional integration’, as defined by the Integra Index, disaggregated for each of the study facilities captured from time-series CFA data. It reveals a significant gap between actual capacity to deliver integrated services (at least one client that day received an integrated package, indicating it was technically possible) and proportion of all visits and all single-provider contacts that were integrated (showing how much the technical possibility was put into practice). Integration within a single provider consultation was generally lower than integration within a visit but mirrored the same pattern indicating that most clients saw only one provider who was able to provide integrated services. This should be caveated because not all clients coming for either an RH or an HIV-related service would necessarily need an integrated service, nevertheless, given the range of services included in the analysis one would expect to see at least some integration of some elements (particularly counselling on HIV testing or FP which would be offered even if the service was not later taken up) and clearly some facilities managed better than others to routinely integrate at least some of their services.

The Figure confirms that Facilities 1 and 14 are the most consistent in providing integrated services over time while Facilities 2 and 21 decline over time. The Figure also presents the availability of non-RH services included in our integration definition (i.e. STI, cervical cancer and HIV services) both within the MCH-FP Unit and within the facility as a whole. Availability of non-MCH-FP services within the Unit is limited in all facilities at both base- and end-line, but availability of the full range of services at the facility level is much higher. By endline there is no difference between the facilities in terms of service availability at either level, yet Facilities 1 and 14 are clearly able to make integrated delivery happen (higher % visits/consultations integrated) while Facilities 2 and 21 are not. To interpret what was happening, we analysed detailed facility-specific data to explore what hardware and software factors might explain these differences in performance.

### Facility case studies: explaining realities on the ground

#### Facility 1 (high-high)

This was a moderately sized rural sub-district hospital. Table 2 shows the client-load and staffing for all facilities at baseline (2009) and endline (2011). In Facility 1 the staff complement increased from 12 clinical staff, two support staff and four casual staff in 2009 to 2013 clinical staff, five non-clinical HIV staff (peer educators and defaulter tracers) one support staff and six casual staff in 2011. Over the same time, the facility doubled its average monthly client load from 485 to 858, with a doubling of FP visits and a huge increase in annual visits for HIV care as it expanded its treatment services.

The facility received support for reproductive health and HIV services from donors, including under a bilateral agreement USAID providing district wide support, as well as regular supplies and funding from central government. Most of the donor support to the facility itself during the study period was for non-clinical support to HIV services: provision of cabinets for drug storage and shelves to keep the files for HIV clients in the MCH unit and refreshments for HIV client support groups. Since 2008 staff have controlled facility spending and seem to manage their resources well, having experienced no serious stock outs or shortages of drugs and supplies (for HIV or FP) or equipment during the study period, even though in interviews staff said they could do with more.

The facility had three rooms in the MCH-FP Unit and a stand-alone CCC (Comprehensive HIV Care Centre) with an HIV counselling and testing (HCT) room, and a 24 h emergency and out-patient department. MCH and HCT were provided Monday–Friday and CCC services for HIV treatment and care officially on 2 days per week although patients were seen any day they came. For pregnant women, prevention-of-mother-to-child-transmission (PMTCT) services were offered on Wednesdays, although women coming at other times were also tested. Nurses rotated quarterly between MCH, wards/maternity, and outpatients. Usually three nurses were assigned to each Department. There were two clinical officers, one of whom worked exclusively in the CCC (with regular visits to MCH for PMTCT).

There was a clear management commitment to moving to department wide HIV testing as well as a model for pregnant women diagnosed with HIV being completely integrated within the MCH unit (until  months post-partum) (Table 2).

Table 3 shows (for all facilities) results from the structured interviews with staff at endline (2011) on their perceptions of the benefits and challenges of integrated delivery. Given the very large increase in clients coming for HIV care to Facility 1 combined with only a modest increase in staff numbers, and external support mainly for non-clinical infrastructure, one might expect perceptions of integration to be rather poor. In general, though, the impression was the opposite. All but one staff interviewed said they thought integration had reduced client visit time at the facility, increased their (staff) awareness of responsibilities and given them a chance to practice more skills than before. More than half thought service efficiency had been improved as a result and, most striking, all staff interviewed said they had experienced improved team work and provider communications after integration. The biggest challenges reported were drug/equipment shortages and lack of staff time—the latter probably a reflection of the doubling of client load over the study period while staff time increased mainly for non-clinical HIV care. Despite the perceived lack of time, staff reported regular supervision and all said they were satisfied with the supervision they received. Further details were obtained from the in-depth interviews.

**Table 3:** Provider perceptions of benefits, challenges and management of integration, at endline (2011)

	Facility 1 (*n* = 7)	Facility 2 (*n* = 6)	Facility 14 (*n* = 5)	Facility 21 (n = 6)
Providers reporting experience of specified benefits				
Reduced client load	0	3	4	4
Cost-effective for the facility	2	1	1	3
Reduced client-visit time at facility	6	5	5	5
Improved efficiency in services	4	3	4	4
Improved team work	7	2	3	1
Improved provider communication	7	6	4	3
Increased awareness of responsibility	6	6	5	4
More skills practiced than before	6	6	4	4
Providers reporting experience of specified challenges				
Occupational stress:				
No occupational stress	2	0	0	1
Has not changed	1	3	1	1
Has reduced	1	1	4	3
Has increased	2	2	0	1
Workload				
Has not changed		1	1	2
Has reduced		0	3	2
Has increased		5	1	2
Shortage of equipment, drugs	4	2	2	2
Shortage of room-space	2	4	3	3
Shortage of staff time	4	3	1	5
Lack of trained staff	2	1	1	1
Lack of clear policies and guidelines	0	1	0	1
Providers reporting experience of management, motivation and performance				
Frequency of supervisory support:				
Once a month (regular)	2	4	3	3
Once a quarter (occasional)	5	2	2	3
No supervisory support	0	0	0	0
I’m satisfied with supervision	7	5	5	5
Level of salary is fair	2	3	5	3
Job conditions do not allow one to perform to high levels	2	5	4	3
Manager consults staff before making job decisions	3	5	5	4

Although only one manager was interviewed in depth, that interview gave the impression of a person who was sympathetic and supportive in demeanour, appreciated her staff and whom staff appeared happy to consult:

*there are some training some staffs have not undertaken and when they come to MCH, they call me for support. I am happy because the providers consult …* (Manager, Facility 1, 2013).

All providers were asked ‘if another organisation was going to integrate services, from your experience, what would you suggest to them on how to go about it?’ In response to this, the senior manager interviewed talked explicitly about the need to support agency and self-confidence in her staff, which can help manage or handle structural deficits (like the lack of rooms) to enable integrated client-based care. She was the only senior staff to talk about supporting staff agency at any point in the interviews across the sites:

*I would suggest to them that even if they have no room, things will flow. […] I would advise them to gain confidence and courage in what they do. This will help them give the best to the patient.* (Manager, Facility 1, 2013).

Integrated delivery is clearly helped by having front-line providers who are willing and able to learn and engage with new skills. The manager interviewed said she had never heard her staff complain about doing more things or having to learn new skills—‘they are willing to learn and to change’. She talked about integration having ‘increased their morale’ and led to improvement in skills.

#### Facility 2 (high–low)

This was a fairly small peri-urban Health Centre in a Provincial town with six clinical staff, one support staff and three casual staff at the beginning of the study, rising to 12 clinical staff, one support staff and four casual staff by the end of the study (see Table 2 for staff FTEs). The facility served a very large catchment area that expanded as boundaries were redrawn, from 23 000 in 2009 to 93 000 in 2011 yet this has little impact on the average monthly client load which only increased marginally from 333 in 2009 to 399 in 2011—presumably because clients continued to use the facilities they had used before the boundary change.

The facility had received support from external agencies for RH and HIV since before the study started. During the study period, two clinical staff (one nurse and one clinical officer) and one support staff (data clerk) were funded by an international NGO to support the CCC (HIV care) work. Like the previous facility staff were in charge of their own spending, but despite external support the facility has suffered from frequent stock-outs of rapid HIV test kits and long-acting contraceptives which are available through the Ministry’s procurement system—suggesting poor facility management.

Emergency and maternity services are provided 24 h, otherwise the facility is open from 8 a.m. to 5 p.m. but usually sees clients between 9 a.m. and 2 p.m. which is when most clients came (and were encouraged to come) because public transport and availability of staff is usually better in the mornings. The MCH unit had two rooms (one for child welfare and one for FP/ANC), a stand-alone HCT room (connected to the OPD); a donor-funded CCC within the facility opened in 2010 (between timepoints 2 and 3, Figure 2) with an additional (donor-funded) clinical officer for the CCC. No FP was provided at the CCC—clients had to enrol at the FP unit, although long-acting methods could be checked at the CCC.

Like at Facility 1 women living with HIV attending the CCC who become pregnant were supposed to be transferred to the MCH unit until the baby is 18 months, but staff said this was not popular with clients (who perceived quality as better in the CCC which is heavily supported by external NGOs) and had led to tensions between the CCC and the MCH Unit:

*the newly diagnosed ones [from ANC] are easier to manage than the ones who are positive and have been to the CCC, since they compare the competency of the two* (Frontline provider, Facility 2).

This tension impedes connections between the two units and seems to have led to resentment among staff at having to see HIV clients in the MCH unit when there is more perceived staffing capacity at the CCC:

*You can have a large queue [here in MCH] and you want to integrate, yet those in CCC can perform the same thing and they do not have [as] many patients as you have, so you end up screening only those who have to be here but compromise for those who can get the service elsewhere* (Frontline provider, Facility 2)*.*

Staff were meant to rotate regularly between services, to keep them multi-skilled, but in practice rotation was limited, partly by religiously-motivated conscientious objection by some staff who refuse to provide condoms or FP services:

*There is a challenge of staff not wanting to change roles and learn new skills particularly when you focus on beliefs and Religion like Catholics. They don’t believe in modern FP methods […] and even issuing of Condoms. […] You therefore find that when they are on duty, they don’t provide these services* (Manager, Facility 2).

The problem of lack of staff able and willing to deliver care seemed to become worse over the study period with high staff turnover at timepoint 3 when integration performance starts to decline very significantly (Figure 2). Three staff moved to Facility 14, which then improved its performance, while replacement staff in Facility 2 seem not to have been a success. One provider noted ‘*it [conscientious objection] wasn’t a problem before’*. One result is heavy workloads for the staff who do provide all services so that ‘*You may end up not giving all the services to the client noting about the time and the workload that you have*’ (Manager, Facility 2) and staff are reluctant to take on extra work:

*now you have to take time with the client through a process of counselling, testing and treatment. Negatively, this has declined the staff morale in that there are those who still feel it when they take a lot of time counselling the client – something that is not so easy* (Frontline provider, Facility 2).

The staff interviews give a very different impression from the previous facility, of a staff in tension, some reluctant to take on new skills, with subsequent frustration among others and lack of mutual support or teamworking. It is notable in Table 3 that only two staff thought teamworking had improved—though surprisingly all interviewed staff thought that communication and awareness of responsibilities had improved since integration. They also recognized that more skills could be practised than previously. Despite a doubling of clinical staff and a virtually static client load staff reported that:

*we normally have only one nurse on duty in the whole facility so integrating becomes a problem […] we are seven nurses, one is on maternity leave, another on annual leave, another on night duty and night off and we are left with two one of whom is on day off and the other is for CCC* (Frontline provider, Facility 2).

This suggests poor management of staff who consequently have limited possibility for effective teamwork or support to share workloads through internal referral between staff. Teamworking was reported to be clearly better before the three nurses left for Facility 14, suggesting they had a significant influence on the way the facility functioned:

*before […] no one nurse was alone, so if the person couldn’t handle something we just called the other and wouldn’t let the opportunity pass, but nowadays she has to postpone if she cannot handle it* (Frontline provider, Facility 2).

Finally, it was noticeable in the interviews with senior staff that they talked about supervision primarily in terms of tools, equipment, commodities and making sure they are all in place. There is no mention of supporting staff to make decisions, work together or discuss problems. Similarly, the front-line providers talk about supervision as simply making sure everyone attended training then implemented the technical skills they were taught.

#### Facility 14 (low–high)

This was another fairly small health centre in a rural location; it was upgraded at timepoint 3 to a sub-district hospital due to a larger district being divided into smaller district units (each needing a sub-district hospital). The upgrading resulted in the creation of more consulting rooms (from an administrative block which was converted in 2012) and an increase in staff—though arguably insufficient to cover all new rooms and take on 24 h duties. Over the study period this facility’s staff numbers were very similar to Facility 2 increasing from five clinical staff, one support staff and one casual staff to 13 clinical staff and three casual staff by 2011. Its average monthly client load in fact reduced significantly from 859 in 2009 to 409 in 2011 (virtually the same endline client load as Facility 2)—this may reflect its reduced catchment population which fell from 40 000 to just 7684 over the same period, with some former clients presumably choosing to go to other upgraded or bigger facilities in their new sub-districts.

The facility has received external structural integration support (resources, equipment and training) and interventions for RH and HIV services from at least five different agencies (mainly implementing partners (NGOs) supported by USAID) though no particular support was recorded during the study period—possibly because it was receiving government support (staff, infrastructure) for its upgrade. The senior staff controlled their spending of government money and seemed to manage forecasting and procurement well, with few reported stockouts during the study.

A staff member was on duty 24 h though the main facility was open between 8 a.m. and 5 p.m. The MCH and child welfare units are integrated with an internal door connecting two rooms: so a woman can go into child welfare and then straight into FP/MCH (where HIV testing is done) without going out again. MCH and VCT were provided Monday–Friday; since 2010 CCC services were provided 1 day per week, usually on a Tuesday, by the Clinical Officer (from the OPD).

Structurally a small facility, staff maximized their limited space through daily coordination and team-working to manage the client flow. The large influx of staff after the facility’s upgrade combined with a very substantial decrease in client load clearly eased workload and would have allowed staff more time to reconfigure to integrate their services. Not surprisingly the staff in the structured survey reported that shortage of staff time was not a challenge, and indeed workload was perceived by most to have reduced, but they remained short of room space although generally integration was regarded as having considerable benefits (Table 3).

In addition to the positive influence of staff numbers on reducing workload and facilitating integration, another feature was that staff reported a collegial way of working which increased during the study period. Indeed, soon after timepoint 3 (Figure 2), when the facility was upgraded, three staff were transferred from Facility 2 (which was a high-performing integrated facility at that point) to the MCH unit in Facility 14—coinciding with an increase in performance here and a concurrent decrease in Facility 2 suggesting these three trained nurses played an important role in both facilities in the way they approached integrated service delivery. By endline it was reported that staff held daily team meetings to decide where they would work and moved daily through units as the client flow required. Nurses rotated between ante-natal, post-natal and FP services, with HIV testing done in all three, as well as between child welfare and maternity services and occasionally the OPD. Indeed during the observational visits for workload analysis the Clinical Officer (in charge) was largely absent and the nurses managed themselves, further highlighting the importance of management and team-working competencies of peer front-line staff.

In late 2009 and late 2010 the MoH put in place ‘rapid results initiatives’—to increase focus on provider initiated HIV testing and counselling over 100 days in the facility catchment area. This may explain the dip in integrated visits/contacts at timepoints 1 and 4 (Figure 2) since at those timepoints most clients would already have tested for HIV in the previous few months meaning counselling and re-testing was not appropriate.

During the in-depth interviews frontline staff reported being happy dealing with HIV services—several spoke of the morale-boost they got since the HIV CCC opened ‘coz if you have a HIV-positive mother getting an HIV-negative baby we get motivated’.

Once again, the answer to the question on what advice to give to other facilities on how to integrate was revealing, showing that staff here were able to internalize integration as an approach to client-management rather than a rigid clinical protocol or set of rules to follow:

*the best way to start with is to make it a part of you, a part of the management of the client who comes here so that they don’t get missed opportunities …* (Frontline provider, Facility 14).

#### Facility 21, (low–low)

This was a large peri-urban sub-district hospital in a suburb of Nairobi. Over the study period its staff numbers more than doubled from 14 clinical staff and two casual staff to 27 clinical staff, seven support staff and 13 casual staff while the average monthly client load reduced very significantly from 1850 in 2009 to 479 in 2011. The baseline catchment area could not be confirmed but by endline it was serving a population of 120 000 although its workload (looking at staff to client load) remained comparable to that in Facilities 14 and 2 and very much lower than Facility 1.

Like the other facilities it received some external support from donors, mainly for FP supplies. Nevertheless, stockouts were frequently reported, including HIV testkit and reagent stockouts regularly throughout 2010 (timepoints 2–3 in Figure 2) and long-acting FP stockouts in early 2010 (between timepoints 1 and 2) and early 2011 (between timepoint 3–4). Observations suggest that this was partly because staff were uninterested and unmotivated to remember to order supplies when stocks were running low.

Staff were on duty 24 h but main facility hours were 8 a.m. to 4 p.m. Monday–Friday and closed Saturday and Sunday. Its MCH/FP unit (providing ante-natal and post-natal services, HCT, FP and child-welfare services) and CCC units were clustered together in one part of the hospital. Rotation of staff appeared to be irregular and there were discrepancies with staff interviewed saying they rotated anywhere between annually and 2–3 monthly.

The facility took a very different approach to HIV counselling and testing, doing it almost exclusively in groups, including self-reading of results, offering virtually no provider input—in contradiction to WHO and MoH protocols. This situation presents a data-problem since the client-flow data on which the Index scores were based would not have picked up the group counselling/testing, thus the scores would inevitably be low. Nevertheless, it is questionable whether group counselling and testing should be recognized as an ‘integrated’ service in the absence of individual provider-led consultation.

The Facility clearly had other problems. Staff interviews reported that the CCC (which in contrast to Facility 2 received no dedicated donor support during the study) was seen by clients as lacking privacy and was hardly used. Staff seemed more focused on integrating FP into other services rather than integrating HIV testing and treatment and staff were reluctant to get involved in HIV services:

*they thought that this [integrating HIV services] was too much for them and they also thought that they were putting themselves at risk* (Manager, Facility 21).

Several staff also mentioned reluctance of some colleagues to provide FP to certain types of clients (young, unmarried) suggesting more judgemental providers which would impede delivery of any sexual and reproductive health related services.

In the structured interviews with staff the results were not particularly negative. It is perhaps not surprising that there seemed to be little negative effect on workload or client load since with the huge increase in staff and concomitant decrease in client-load staff had more time and worked at considerably under-capacity, at least on paper. The in-depth interviews with six frontline staff, however, gave a strong impression of negative working experiences, poor teamworking, communication or support. Two issues in particular emerged. First, front-line staff felt they were having to take on the work of the doctors who were not present for much of the day. The doctor responsible for maternity and theatre lived in a different town and only came occasionally, so:

*… being a nurse we are forced to be clinicians since there are no clinicians so this makes your work high’* (Frontline staff *Facility* 21).

Second, there were clear unequal distributions of work during the day, with the majority of clients coming in the mornings (for a variety of reasons) leading to exhaustion and perceived over-work even when afternoons were very quiet:

*… there was a time when us health workers used to ask the clients to come in the morning, and nobody should come in the afternoon, and that notion is so much deeply rooted in the clients mind and to wipe it out will take some time […] others will come in the morning due to mode of transport; others […] they know that they can only get doctors in the government hospitals in the morning* (Frontline staff, Facility 21).

Overwork leads to omission of services and staff reported missing out HIV testing and sexual behaviour assessment if they were too busy. Overall the frontline staff came across as demoralized and feeling unsupported but lacking the motivation (or perhaps agency) to change the situation. It is not surprising that, like at Facility 2, supervision seems rigidly structural rather than addressing issues of collegiality, workload-sharing and decision making:

*we look at the documentation and whether the commodities have been utilised in the proper way* (Manager, Facility 21).

## Discussion: explaining good integrated performance over time

The Integra Index (Figure 1) shows the two well-performing facilities over time (Facilities 1 and 14) in fact had lower structural integration (systems hardware) scores than the two facilities that performed poorly over time (Facilities 2 and 21), yet were able to more consistently deliver integrated services to their clients.

Quantitative facility data indicate some important differences between facilities in terms of case-load and staffing. In Facility 1 (high–high) both staff complement and client-load doubled over the study period. In contrast Facility 14 (low–high) also doubled its staff but saw a halving of its client visits (across all services except cervical cancer which was not offered at baseline) largely due to a change in its catchment boundaries (reducing its catchment population by two-thirds). This significantly reduced the workload for staff which must have contributed to its improvement in integrated delivery over time. Nevertheless, at endline Facility 14 has lower staff numbers than Facility 2 (high–low) and Facility 21 (low–low) yet a similar client load, suggesting staff at Facilities 2 and 21 were less able to cope. The distribution of staff across services also differs between the poorer and stronger performing facilities with staffing better matched to client-load across the services in the better performing facilities. Overall these data suggest that while in numerical terms staffing may play a role in explaining integration performance, other factors are clearly involved.

Structured interviews with staff suggest there is better experience of teamwork at the better performing facilities, further, the contextual and qualitative data underline that the management of staff organization, distribution and working practices as well as supportive and collegial team-working play a critical role and can influence how well staff are able to make use of the structures and resources they have. In other words the systems software (people) can affect how effectively the systems hardware is utilized to deliver integrated services.

In our study, some common factors seem to emerge. Better performing facilities (1, 14) seem to have: better connections between the CCCs (HIV care) and the MCH/FP unit; managed stock forecasting well so experienced no/few stockouts; have regularly rotating staff who are well distributed across the different services; staff eager to learn and implement an expanded skills set; motivated staff able to work supportively in teams to manage changing client flow; managers who understand supportive supervision to be about enabling their staff to gain confidence and experience in making decisions about what the client in front of them needs as well as ensuring they have the equipment and supplies to do their work.

Conversely, the poorly performing facilities (2, 21) show an inability to prevent stockouts despite all facilities now having control over their income, poor management and inappropriate distributions of staff with frequent absenteeism and infrequent rotation leading to an inability to manage high-workloads or support team members. These facilities seemed much less able than the other two facilities to absorb the routine disruptions caused by Ministry of Health’s policy to rotate many staff every two years and their creation of many new districts which creamed off senior frontline staff to head up new district administrations during the study period. It is notable that in the two poorly performing facilities supervision was approached in an instrumental manner, as a tick-box exercise relating to whether commodities or protocols were being correctly utilized (which is what many donors continue to require). Staff reluctance or refusal to provide services (especially FP) on moral or religious grounds was also a particular problem in these facilities and compounded staff shortages and high workload. Another issue was very evident in Facility 2 which was the impact of vertical donor support to the CCC (HIV care) services which created an inequity in service-quality and a tension between staff in the MCH unit who were supposed to look after pregnant HIV clients but felt they were undermined by the CCC staff whom clients regarded as ‘better’ and who were seen to have more time.

**Figure 3:**
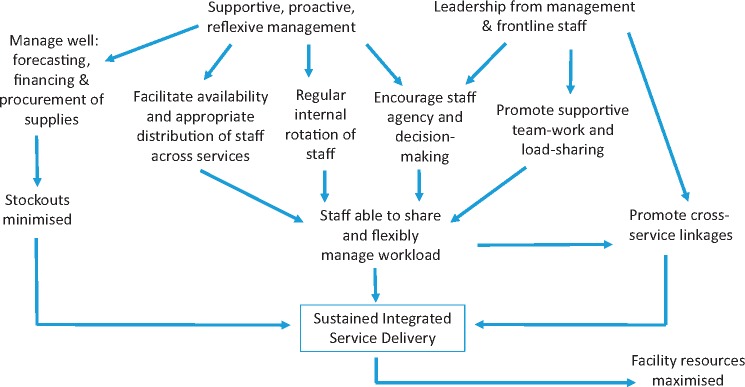
‘People’ factors influencing successful delivery of integrated services

Overall, our findings suggest that structural factors like stock-outs, distribution of staffing and workload and rotation of staff as well as the way external support is given, continue to affect ability to provide integrated care. Nevertheless, our studies also show that these factors can be influenced at least to some degree by the staff themselves. Our case studies suggest that the ability of some staff to overcome some of the structural deficiencies is dependent on excellent leadership (at management and frontline level) as well as motivation and agency among frontline staff to share workload and work flexibly to support each other. Figure 3 visualizes this.

Structural inputs are clearly important, but without due attention to the support given to staff to ensure they are motivated and encouraged to make their own decisions and to work together to share workload, the impact of structural investments will be limited. With good management, for example, stockouts may be minimized, use of facility resources maximized and, most importantly, staff supported to make decisions to share and flexibly manage their workloads. A study in Zimbabwe found that even where health workers in remote areas had few financial incentives and hard working conditions, where they had good leadership and supportive management they still exhibited a high level of motivation to perform well (Stilwell 2001).

But there is a caveat: there should not be an expectation that staff ‘should’ be able to overcome structural deficiencies including lack of equipment, drugs or trained staff. Rather the expectation should be a commitment from Ministry and donors to recognize and invest in both the necessary resources and materials ‘and’ good leadership and management, with the latter playing an important role whether or not there are resource shortages. Where there are shortages, good leadership and management can help to ensure structural investments are used as best they can be and to support and encourage frontline staff to feel they can make a difference even when they are working in difficult conditions. There is an established literature, particularly in the field of nursing, on the importance of leadership and management for staff retention, motivation and performance (Drach-Zahavy 2003, 2004; Heller *et al.* 2004; Willis-Shattuck *et al.* 2008). An international systematic review found that studies reporting leadership styles that were focused on people and relationships were associated with higher job satisfaction, while studies reporting leadership styles focused on tasks were associated with lower job satisfaction (Cummings *et al.* 2010). Our findings support this with staff in the two highly performing Facilities demonstrating an understanding of supervision being much more about supporting staff to work together to do their jobs while in the poor performing Facilities staff talked only about tasks and equipment.

Notions of ‘trust’ in management-provider relations have also been recognized as important (Gilson *et al.* 2005; Gilson 2006). In a recent review of health worker motivation and trust relationships (Okello and Gilson 2015) most of the studies that showed a positive impact of colleague-trust relationships. Notably, all the studies showing positive impact of supervisory relationships were from high-income settings, while all examples of negative experiences of supervisory roles were from low-income settings. Integra provides some important findings on positive workforce relationships in low income settings. In our study, the relationship of trust between colleagues appears to be at least as important as that between frontline workers and managers. Individuals, who were able to work as a team with peers and could support each other emotionally, as well as be valued by their superiors and engaged in decision-making processes, were better able to provide integrated care to their clients. The influence of the group of nurses who moved from facility 2 to 14 very clearly illustrates that a strong team of frontline staff can help mitigate poor or indifferent management and structural deficiencies.

There are a number of research, policy and practice implications that arise. First, there is a need for health systems research to develop, test, then scale-up interventions to promote the development and sustaining of ‘agency’ and team-working among frontline staff. Second, commitment and accountability is needed from donors, policy, and senior management staff to support such interventions to deliver responsive, integrated care.

## Conclusions

The Integra case study findings presented here have demonstrated that structural integration is not a sufficient condition for integrated service delivery. Although the numbers and the structures tell you what might be possible, it is the people in the system who enable integration to happen. Our case studies show that in some cases excellent leadership and peer-teamwork has enabled facilities to perform well despite resource shortages. Nevertheless, resourcing and mid-level governance deficiencies are likely to inhibit integration in other facilities where leadership will remain average and teamwork more transactional. Far more attention therefore needs to be paid to how to promote sensitive management of staff to nurture and support their agency in decision making, team-working and load-sharing as far as possible to enable staff to work flexibly to meet the challenges that face providers each day. This ability to provide an integrated or joined-up package of services to meet changing needs becomes even more relevant as health systems face changing constellations of chronic and non-communicable as well as infectious diseases and changing disease burdens as a result of climate-change. There is a long way to go to understand how best to nurture and promote supportive management and front-line team-work in low-income settings, but support it we must if health systems are to be sufficiently resilient to meet future challenges with confidence.

## Ethics

Ethical approval was granted by the Kenya Medical Research Institute (Reference: NON/SSC/113 and 114); the Research Ethics Committee of the London School of Hygiene and Tropical Medicine (approval number 5426), and the Population Council institutional review board (approval numbers 443/444). The Integra Initiative is registered with Clinical Trials (ClinicalTrials.gov) as NCT01694862.

## Footnotes

1 In 2013 the Government of Kenya undertook a realignment of its Provincial boundaries and Provinces became ‘Counties’ in the new configuration. This was after fieldwork had been completed therefore we retain the designation that was in place at the time of fieldwork.
